# Combined logical and data-driven models for linking signalling pathways to cellular response

**DOI:** 10.1186/1752-0509-5-107

**Published:** 2011-07-05

**Authors:** Ioannis N Melas, Alexander Mitsos, Dimitris E Messinis, Thomas S Weiss, Leonidas G Alexopoulos

**Affiliations:** 1 Dept of Mechanical Engineering, National Technical University of Athens, 15780 Zografou, Greece; 2 Dept of Mechanical Engineering, Massachusetts Institute of Technology, Cambridge, MA 02139, USA; 3 Center for Liver Cell Research, Department of Pediatrics and juvenile Medicine, University Medical Center Regensburg, Regensburg, Germany

## Abstract

**Background:**

Signalling pathways are the cornerstone on understanding cell function and predicting cell behavior. Recently, logical models of canonical pathways have been optimised with high-throughput phosphoproteomic data to construct cell-type specific pathways. However, less is known on how signalling pathways can be linked to a cellular response such as cell growth, death, cytokine secretion, or transcriptional activity.

**Results:**

In this work, we measure the signalling activity (phosphorylation levels) and phenotypic behavior (cytokine secretion) of normal and cancer hepatocytes treated with a combination of cytokines and inhibitors. Using the two datasets, we construct "extended" pathways that integrate intracellular activity with cellular responses using a hybrid logical/data-driven computational approach. Boolean logic is used whenever a priori knowledge is accessible (i.e., construction of canonical pathways), whereas a data-driven approach is used for linking cellular behavior to signalling activity via non-canonical edges. The extended pathway is subsequently optimised to fit signalling and behavioural data using an Integer Linear Programming formulation. As a result, we are able to construct maps of primary and transformed hepatocytes downstream of 7 receptors that are capable of explaining the secretion of 22 cytokines.

**Conclusions:**

We developed a method for constructing extended pathways that start at the receptor level and via a complex intracellular signalling pathway identify those mechanisms that drive cellular behaviour. Our results constitute a proof-of-principle for construction of "extended pathways" that are capable of linking pathway activity to diverse responses such as growth, death, differentiation, gene expression, or cytokine secretion.

## Background

Construction of signalling pathways is a major endeavour in biology. Signalling cascades, starting at the receptor level, orchestrate a variety of normal or pathological responses via a complex network of kinases, adaptor molecules, and other signalling proteins [1]. Several gene- and protein-based approaches have emerged for elucidating the complex intracellular signalling activity. Gene-based analysis has the advantage of whole genome exploration [2-4] whereas proteomic approaches are applicable on small pathways but with a more reliable view of pathway function, since proteins are the ultimate reporters of cellular activity [5,6]. Both approaches aim at a holistic understanding of cellular actions; that is how to link the environmental cues to the intracellular signalling activity and then to cellular response [7,8].

Several types of computational models have been proposed to elucidate the complex intracellular signalling network and are commonly classified as data- or topology- driven methods [9,10]. Their main conceptual difference is their methodology for identifying intracellular connectivity: data-driven models are highly abstract and can identify molecular dependencies within experimental data based on regression analysis, i.e., principal component analysis-(PCA), Partial Least Square Regression (PLSR), Multi-Linear Regression (MLR), Bayesian or other probabilistic models [11-14]. On the other side, topology-driven models rely on *a-priori *knowledge of the signalling connectivity and depending on their signal-propagation assumption are classified as physicochemical, fuzzy, or logical. In physicochemical models signalling events are modeled via chemical reactions using ordinary or partial differential equations (ODE or PDE) depending on their ability to model spatial gradients of signalling molecules [15]. Despite their detailed representation of the transduction mechanisms, ODE or PDE -based approaches require a large number of parameters, i.e. reaction rate constants and initial conditions, that makes them practical to very small pathways such as the EGFR pathway [16]. To overcome that limitation, fuzzy models have suggested a simplified -but continuous- representation of the transduction mechanism, which can be applicable to medium-to-large topologies [17,18]. On the other side of the topology-driven spectrum, logical models are based on a simplified (on/off) representation of the signalling transduction mechanism and thus, are applicable to very large topologies [19-22]. Logical models derived from canonical pathways have several mismatches with phosphoproteomic measurements [20] and thus, a genetic algorithm or an Integer Linear Programming formulation have been developed to construct cell-specific topologies and identify drug-induced pathways alterations [18,23,24].

Even though most experimental data conform on a *Cue-Signal-Response *(CSR) paradigm [25,26] most of models -apart from limited cases [18,27]- are capable of representing events from either cue-to-signals or from signals-to-responses: topology-driven models are applicable on cue-to-signal datasets where a significant body of literature allows the construction of canonical maps, where data-driven models are applicable on signal-to-response datasets where the flow of information is not fully understandable. Thus, currently there is a lack of models that can answer how stimuli via their signalling mechanisms orchestrate diverse cellular responses such as gene expression, migration, growth, death, metabolic activity, or cytokine release.

In this paper we present the construction of "extended" pathway models that aims to explain cellular responses based on pathway activity. The main idea behind the computational approach is a hybrid Boolean/data-driven model where a logical model is used whenever *a priori *knowledge is accessible and a data-driven approach is used for adding non-canonical edges to reach out to cellular responses. A previously developed integer linear programming (ILP) framework [23] is modified to incorporate non-canonical edges with weights that correspond to regression coefficients and used to optimise the connectivity of the hybrid pathway. The resulting pathway is capable of linking signalling pathways to any type of quantifiable readout such as measurements of cell growth, necrosis, apoptosis, cytokine secretion, or transcriptional activity, as long as these data are available under the same experimental conditions as the phosphoproteomic dataset. As a case study, we construct extended pathways for studying hepatocellular carcinoma (HCC), a liver cancer disease that is the third leading cause of cancer death with inadequate therapeutic interventions [28,29]. As cellular response we choose the release of 22 cytokines and we ask what signalling activity downstream of 7 receptors, and 57 signalling molecules can explain the complex profiles of cytokine releases. Our computational approach is able to uncover well-known secretion pathways and identify significant differences between non-HCC and HCC cells. Our approach highlights the importance for construction of integrated CSR pathways that given a specific stimulus, can predict the intracellular activity that drives responses such as growth, death, differentiation, gene expression, or cytokine secretion.

## Results and discussion

### Construction of CSR Datasets

**Figure 1:**
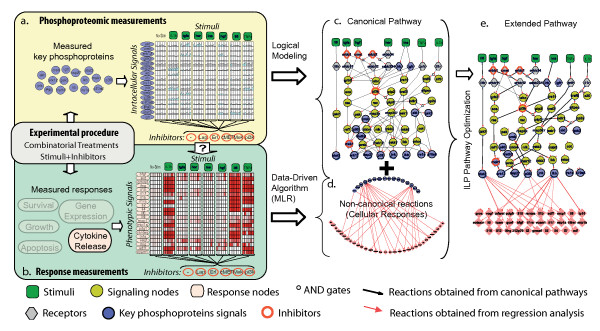
**Experimental and Computational workflow**: (a) The "signalling" dataset monitors the activity of 16 different key phosphoproteins (blue nodes) under the combinatorial treatment of stimuli (green nodes) and inhibitors (red circles). (b) The response dataset can include any quantifiable cellular response such as cytokine releases (22 pink nodes) that are monitored under similar treatments. (c) A canonical pathway that incorporates the stimuli and key phosphoproteins is constructed from the literature (d) Data-driven approach is used to connect the signalling nodes via "non-canonical" edges to cytokine releases. (e) Canonical and non-canonical edges that fit the experimental data are selected using an ILP optimisation formulation and thus, the extended pathway topology is constructed.

**Figure 2:**
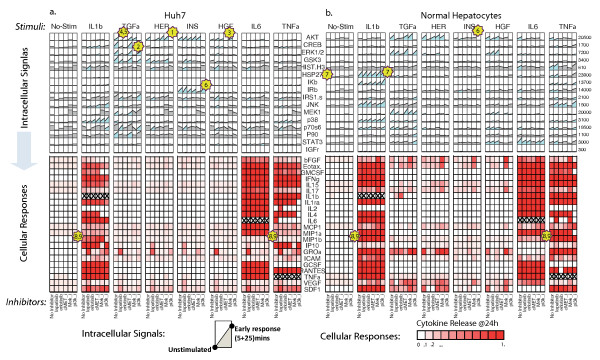
**Experimental dataset in (a) Huh7 hepatocellular carcinoma cell type and (b) primary hepatocytes**. Top panels correspond to the signalling dataset. Each small subplot consists of two datapoints: the zero "unstimulated" condition and the "early response" which is the average phosphorylation activity at 5 and 25 minutes post-stimuli treatment. Bottom panels correspond to the response dataset where 22 cytokines were measured 24 hours post-stimuli. The red colour intensity is proportional to the percent increase of the cytokine release as compared to the basal (unstimulated) condition.

For the construction of the extended pathways, a CSR dataset is created using the beads-based ELISA assays of xMAP technology (Luminex, Austin, TX) as described in the experimental setup (see Material and Methods) and shown in Figure 1. Our experimental data consists of the signalling subset (phosphoproteomic data) and the response subset (cytokine releases) that were measured via multi-combinatorial treatments on two cell types: primary hepatocytes and a hepatocellular carcinoma cell type known as Huh7 [30]. Approximately 50 different perturbations are imposed to primary and HCC cells created by the combinatorial treatment of 7 diverse stimuli (+ no stimulus treatment) and 5 inhibitors (+no inhibitor treatment). As pro-growth stimuli, Tumor Growth Factor alpha (TGFa), Hepatocyte Growth Factor (HGF) and Heregulin (HER) have been chosen based on the response yielded on liver cells in previous experiments [13]. Interleukin 6 (IL6), IL1b and Tumor Necrosis Factor alpha (TNFa) have been chosen as inflammatory ligands. In addition, the Insulin (INS) pathway has been included because of its major role in liver homeostasis [31]. To better constrain the optimisation of pathways we impose additional perturbations using stimuli in combination of selective and potent inhibitors for MEK, PI3K, cMET, and EGFR/ERBB2 Lapatinib and Erlotinib [32-34]. For each combination of stimulus and inhibitor, the phosphorylation state of 16 key intracellular proteins and the release of 33 cytokines were measured as detailed in Materials and Methods section and presented in Additional Files 1 and 2. Among the cytokines, 22 showed a significant activity in either primary or Huh7 hepatocytes. These are plotted in Figure 2 using the DataRail software [35].

Several interesting signalling features can be observed simply by inspection of the data. As positive control observations, all inhibitors block their nominal downstream targets proving their potency and indicating an error-free execution of the multi-combinatorial pipetting procedure (see numbered stars in Figure 2; star#1:PI3K inhibitor blocks AKT under any treatment, star#2:MEK inhibitor blocks ERK under any treatment, star#3:cMET inhibitor blocks AKT under HGF, star#4:Erlotinib blocks AKT under TGFα, star#5:Lapatinib blocks AKT under TGFα). In addition, significant differences can be observed between the two cell types: Huh7 cells respond stronger to insulin stimulus by activating the pro-growth signal AKT and their receptor IRβ compared to primary cells that remain unaffected (Figure 2, star#6). Furthermore, the basal and IL1b -induced phosphorylation activity of the pro-stress protein HSP27 is higher in hepatocytes (Figure 2 star#7). With respect to cytokine data, primary cells appear to respond stronger under inflammatory stimuli by releasing the inflammatory cytokines MIP1α and MIP1β under TNFa and IL1b treatment, an observation that has been seen before as a mechanisms for HCC cells for evasion of immune surveillance (Figure 2 star#8, star#9). Even though significant differences can be observed simply by visual inspection of the data, the main question remains on how the cytokine release profile (bottom panels in Figure 2) can be explained by the pathway activity (upper panels in Figure 2). An answer to this question is the presented methodology for construction of extended pathways that incorporates the pathway activity as well as the cytokine release outcome.

### Computational Framework

The construction of extended signalling pathways can be divided into three main steps: (a) the construction of canonical pathways, (b) the identification of new edges between signals and response from data-driven algorithms, and (c) the optimisation of the extended pathway using an Integer Linear Programming (ILP) formulation.

**Figure 3:**
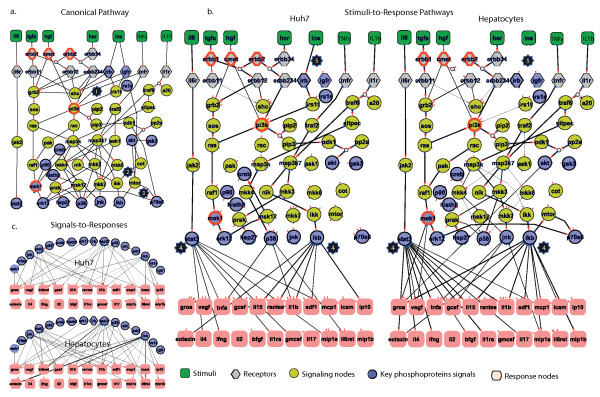
**CSR pathways for primary non-HCC hepatocytes and HCC (Huh7) cell types**. (a) Generic pathway comprised of canonical edges extracted from literature (b) non-canonical edges for Huh7 and primary hepatocytes extracted from a data-driven approach (multi linear regression) (c) extended pathways for Huh7 and primary hepatocytes constructed by fitting canonical and non-canonical edges to experimental data via an ILP formulation.

The canonical pathway map (Figure 3a) is created around the 7 stimuli and the 16 key phosphoproteins using Ingenuity software (Redwood City, California) and manual curation based on literature search [23]. Non-canonical edges (Figure 3c) from key phosphoproteins to cytokine releases are then added to the generic topology and incorporated into the ILP objective function using stoichiometric representation with weights (in chemical reactions these are usually referred as "yields") that equal to the regression coefficients obtained from a multi linear regression (MLR) algorithm (see also Material and Methods for the detailed formulation). This strategy allows us to enhance the canonical topology with response nodes using non-canonical edges from data-driven algorithms that have as dependent values (**Y**) the cellular response and as independent values (**X) **the key phosphoproteins nodes. With this strategy, any type of data-driven approach can be merged with canonical pathways. Herein, MLR was chosen because of its simplicity to connect signals to response in an intuitive way and without the need of intermediate nodes (e.g. nodes representing principal components if PLSR had been used).

Once the extended topology is created with canonical and non-canonical edges, an optimisation formulation with binary variables and linear constraints is employed to identify a pool of pathway solutions that best describes the proteomic data. The main concept behind the ILP optimisation is the minimization of an objective function that represents the deviation between the experimental measurements and the signalling and response values inferred from the network topology, penalized by a function of the map's size. Raw data were normalized to [0,1] as described previously [24] by taking into account the experimental noise, the saturation limits of the assay, and the basal level at time zero (see also Additional File 3, S1: Data Normalization). There are three main terms in the ILP objective function as detailed in Materials and Methods:(1)

The first term penalizes the measurement-prediction mismatch of the key phosphoproteins and removes all edges that contradict the "signalling" dataset. The second term penalizes the measurement-prediction mismatch of the response measurements and prunes non-canonical edges that contradict the response dataset. The third term removes all edges that have no effect on the measurement-prediction error and thus penalizes the map size.

The ILP formulation is solved with the state-of-the-art commercial code CPLEX through GAMS [36,37]. This solver guarantees minimal error between experimental data and the Boolean topology eliminating uncertainty associated with heuristic methods such as genetic algorithms. To overcome the existence of multiple near-optimal solutions, in the present work the ILP solver furnishes 100 distinct solutions within a 10% difference in the objective value. The resulting pathways are presented in Figure 3b where the width of each edge corresponds to its frequency in the pool of near-optimal solutions.

### Model Validation

To evaluate the performance of our hybrid model, several *in-silico *tests were performed including comparison with a data-driven (regression) model (Additional File 3, S2a/b) and assessment of model sensitivity to i) optimisation parameters, ii) experimental design, iii) data deterioration, iii) generic topology. Detailed validation data can be found in the Additional File 3 (S2 and S3) but key points are highlighted in the following section.

#### Construction of a 2-step Multiple Linear Regression (MLR) model and comparison to our approach (Additional File 3, S2)

The performance of our hybrid ILP-MLR approach is compared against a data-driven 2-step MLR approach [13] that correlates i) stimuli and inhibitors to the measured phosphoproteins and ii) phosphoprotein activities to cytokine releases. The two methods are compared quantitatively for data fitting and qualitatively for capturing biological insight. Quantitatively, the 2-step MLR is an unconstrained approach and as such, fits the experimental data better than the ILP approach as indicated by the measurement - prediction mismatch (12% for 2-step MLR, 18% for the hybrid ILP-MLR approach). Qualitatively, the ILP approach predicts the optimal topology based on the canonical pathway and as such, is better in uncovering protein connectivity that is supported by the literature. In contrast the MLR approach can uncover correlations that lack biological interpretation, such as Lapatinib induced IRB, MEK1, HSP27 and P70S6 activation (see Additional File 3, Figure S3).

Overall, most differences in the performance of the two methods come from the different perspectives they adopt in constructing signalling pathway. 2-step MLR is a data driven approach [13,15] aiming at correlating inputs to outputs ignoring any *a-priori *knowledge of protein connectivity. On the other hand, the ILP approach attempts to take advantage of the *a-priori *knowledge in the literature and make a canonical topology to comply with the proteomic data.

#### Assessment of model sensitivity to changes in experimental design (Additional File 3, S3.1)

It is apparent that the optimised topology is based on the experiments performed, the number of signals that were measured, and the number of perturbations imposed in the network. More specifically, a single stimulus experiment with only one measured signal can provide information for a very small subset of the generic topology, and as such the optimised map will be very small. On the other hand, an extensive experiment with all different combinations of stimuli and inhibitors is not possible due to time and cost limitations. In this study, we created a dataset that is experimentally feasible and includes all possible combinations of single stimulus with single inhibitor. Removal of 50% of these treatments randomly shows a significant deterioration of the constructed pathway and 35% increase of the fitness error (see Additional File 3, S3.1). Finding an optimal experimental design for maximally constraining a generic topology is a very important aspect in the field of pathway optimisation that can include pathway controllability, pathway observability, experimental limitations, and definitely several other experimental constraints imposed by how the assays are performed.

#### Assessment of model sensitivity to changes in generic topology (Additional File 3, S3.2)

Despite the wealth of information found for pathway construction mainly from pathway databases, conflicting reports in pathway connectivity makes the construction of the generic topology a non-trivial task with significant manual curation and with no guaranteed for the "right" generic topology. In order to assess the sensitivity of our hybrid model to changes in the generic topology, we substitute up to 10% of our generic reactions with random reactions. As expected, the optimised pathways are highly dependent on the generic topologies and with errors that can go up to ~90% when 10% of reactions are substituted. A possible way to reduce the sensitivity to the generic topology is to allow the addition of less known or conflicting reactions with weight based on literature findings. However, such a method should be coupled to a text mining approach and a pathway database which is beyond the scope of this study.

#### Calibrating the weights of the three objective terms (Additional File 3, S3.5)

The two prediction mismatch terms were given equal weights(= 1). In contrast, for the map-size reduction term a significantly smaller weight was selected (= 1/20). This weight was chosen based on the longest chain of consecutive reactions, namely 12, with the purpose to force the optimiser not to remove edges if they are essential for satisfying experimental results. For example, consider a chain reaction R1→ R2→...→ R12 that should be kept because experimentally we found the relation "R1 = 1 implies R12 = 1". The reward for the optimiser to satisfy this chain reaction should be more than the penalty that it has to pay for keeping all 12 reactions. Therefore, if by keeping all reactions the map size reduction term increases the objective function by 12 units, then the reward for satisfying a chain of 12 reactions (mismatch term) should be higher than 12. The maximum chain in our pathway is 12 reactions but we choose 20 in case that further refinements in the generic topology increase the maximum chain.

### Construction of signals-to-response pathways

The generic map includes a total of 7 receptors, 57 signalling molecules connected with 113 canonical edges, and 352 non-canonical edges that connect the 16 key phosphoproteins to the 22 cytokines. From the 352 non-canonical edges, a large percentage of those have correlation weights close to zero. To minimize the computational cost of the ILP solution, we choose to retain 60% of those weights as explained in the Additional File 3, S3.4. Extended topologies were created for non-HCC and HCC (Huh7) hepatocytes. The mismatch between generic pathways and non-HCC or HCC datasets is 41.0% and 46.6% respectively (see Materials and Methods for definition of error). After optimisation, a total of 47 canonical and non-canonical edges remained in Huh7 and 43 in non-HCC hepatocytes. The error of the cell-specific pathways drops to 18% in Huh7 and 17% in non-HCC hepatocytes. Several edges are removed due to conflict with the data. One example is the removal of TNFR → PI3K edge in both cell types in order to isolate the AKT and MEK activity from the TNFa stimuli (star#1, Figure 3). In a similar manner the AKT→ COT→ IKK→ IKB edges are removed because the measured AKT and IKB signals are not co-regulated as suggested by the Boolean logic (i.e., AKT = 1 then IKB = 1) (star#2, Figure 3). Furthermore, the links for activating p70S6 on a PI3K independent manner remain only on the primary hepatocytes as suggested by the dataset (IL1b and TGFa activates p70S6 with or without the presence of a PI3K inhibitor, star#3, Figure 3).

The presence of cellular response data significantly enhances the optimisation of the signalling topology in two different ways. Firstly, non-canonical edges provide additional pathway information to the ILP formulation. In other words, the optimiser is forced to conserve edges that lead to cytokine nodes but do not affect measured phosphoproteomic signals (see "Impact of response measurements on pathway optimisation" in Additional File 3, S4). Secondly, edges with marginal activations of intracellular signals that otherwise would be considered insignificant (either because of assay limitations or time-point selection) are retained in the topology if they correlate well with cellular response. An example of this case is the IL6 pathway: although IL6 activation of STAT3 in Huh7 is seemingly undetectable (see raw signalling data in Figure 2), the IL6→ ...→ STAT3 pathway is conserved because even small chances in the STAT3 activation levels correlate well to the IL6-induced release of various cytokines (see star#4 in Figure 3) (see "Impact of cellular response measurements on pathway optimisation" in Additional File 3, S4). Taken together, when the ILP formulation uses both the phosphorylation and response data, it conserves pathways with barely detectable signalling activity as long as they correlate to cellular response.

The non-canonical edges in Figure 3 show that major pathways for the release of inflammatory cytokines are the IL6→ STAT3, IL1b→ NFkB/p38, and TNFa→ NFkB/p38 pathways [38]. Just three key phosphoprotein signals (STAT3, Iκβ and to a lesser extend p38) are responsible for the release of most inflammatory cytokines including TNFa, GROa, RANTES, MCP1, ILb, and EOTAXIN (star#4, Figure 3), an observation that is in accordance to a large body of literature [39]. It is less known how many different pathways can lead to the release of a particular cytokine. A simple enumeration of paths that lead to cytokines for primary hepatocytes (Figure 3c), shows that more than 50% of the cytokines are induced by 2 or 3 edges that can be activated by up to 3 different stimuli following at most 3 different routes of activation. Since the constructed pathways are small subsets of the actual pathways, it is obvious that the mechanisms for a single cytokine secretion are numerous and complex. To tackle such complexity, graph theory analysis of the extended pathways (always limited by the lack of experimental approaches to decipher the whole signalling network) can identify central nodes or group of nodes for inhibiting cytokine secretion, and thus, increase the efficacy of pharmaceutical interventions. This is in particular applicable for multi-targeting of STAT3, NFkB, or p38 pathways to achieve anti-inflammatory effects, a major endeavour of pharmaceutical industry with significant investments on mono-targeted approaches for STAT3, NFkB, or p38 on several diverse diseases including p38 for rheumatoid arthritis [40], IκB for airway inflammation [41], or STAT3 and NFkB for HCC [39,42,43].

#### Independent experimental validation of the model

**Figure 4:**
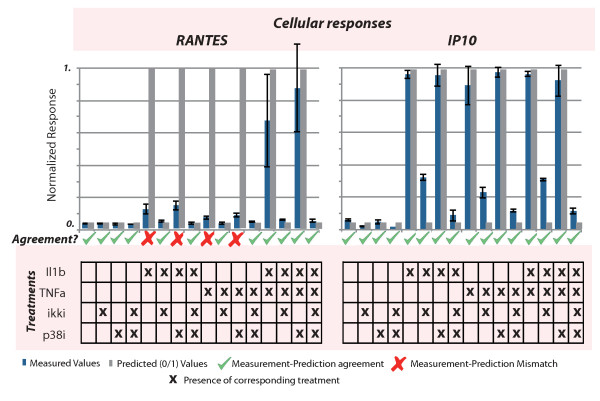
**Validation of the hybrid model predictive power and evaluation NFkB and p38/HSP27 pathways in the release of RANTES and IP10 cytokines in Huh7 cells**. Cytokines were measured upon combinatorial treatments of IL1b, TNFa stimuli and inhibitors for ikk (ikki) and p38/HSP27 (p38i). Agreement between hybrid model and experimental results is denoted with YES/NO symbols.

In order to evaluate the predictive power of our hybrid model, we asked how well the Huh7 model shown in Figure 3b captures the correlation of cellular response to phosphoprotein activity. To achieve that, we choose the pathways IL1b/TNFa to P38/IKB that play major role in cytokine secretion, we block them with potent and selective IKB and P38 inhibitors, and we ask how well our model can predict the IP10 and RANTES, two major players for cytokine release. Figure 4 shows the experimental results and the mismatches with the hybrid model. Our hybrid model was able to recapitulate the IP10 release upon introducing IL1b, TNFa or both in an IKK dependant but p38/HSP27 independent manner. On the other hand, the hybrid model did not fit the induction of RANTES upon IL1b or TNFa stimulation probably not because there was no induction (an almost two fold trend can be seen in the IL1b induced RANTES) but because the induction does not pass the 0.5 threshold so the logic model to consider it an "ON" event. This issue highlights the importance of data normalisation: currently data are normalised to the maximum cytokine value among all treatments. In the follow up experiments, one treatment is the combination of IL1b and TNFa where Huh7 cells show a super-induction of RANTES and makes all other RANTES values to be considered low. Logic models cannot handle such non-linear behavior and lead to predictive errors. When Huh7 treated with the combination of IL1b and TNFa then the hybrid model was able to perfectly recapitulate the RANTES release in an IKK dependent and p38/HSP27 dependent manner.

## Conclusions

In the present work, we developed a method for linking signalling data to cellular response. As a case study, we compare extended signalling topologies of primary hepatocytes and Huh7. The two pathway maps are significantly different. Huh7 are not as responsive as primary cells since only 17 non-canonical edges exists in Huh7 compared to 28 in primary hepatocytes (see also Additional File 3, S5 for a comparison of simulation runs for the two cell types). These findings are in agreement with recently published data that shows HCC cell types are less responsive to Toll Like Receptor (TLR) stimuli than primary hepatocytes [13], presumably to avoid detection and clearance by the innate immune system [44-46]. Major pathway differences related to a survival advantage for HCC can also be observed at the intracellular level: a closer look into the insulin pathway shows that INS→ IRβ and INS→ IGFR edges are removed in hepatocytes but the INS→ IRβ is retained in Huh7 (star#5, Figure 3). A closer look into the raw data (Figure 1) shows that insulin barely induces IRβ and AKT in primary cells. This is in accordance to recent findings that shows increased AKT activation correlates well with the formation of liver tumours [47,48]. However, in that study, the authors pinpoint the mechanisms of AKT overactivation to the reduced expression of p85α - a regulatory subunit of PI3K. Herein, we show that -at least for the Huh7 case- diminished Akt activation levels can be due to receptor's lower activation as shown from the phosphorylation of IRβ.

Here we presented a method for constructing extended pathways that start at the receptor level and via a complex intracellular signalling pathway identify those mechanisms that drive cellular response. Because of the nature of response data - where detailed mechanisms are sparse and not easily searchable via text mining approaches- we used a data-driven approach to link intracellular activity to cellular responses via non-canonical edges. Those edges, together with well-defined intracellular pathways, were used for the construction of the "generic map" which is finally optimised to match high-throughput protein data. The resulting extended pathways revealed intracellular mechanisms that are responsible for the release of 22 cytokines and correlate well with a large body of literature that pinpoint at STATs and NFκB as major drivers of inflammatory stimuli. More importantly, comparison between cell types shows significant differences that lead to survival advantages of the HCC cells. Our results constitute a proof-of-principle for construction of "extended pathways" that are capable of linking pathway activity to diverse responses such as growth, apoptosis, differentiation, gene expression, or cytokine secretion.

## Methods

### Experimental procedure

Primary human hepatocytes were isolated and cultivated in serum-free Williams' Medium E (Biochrom AG, Berlin, Germany) [49]. The viability of isolated hepatocytes was determined by trypan blue exclusion. Only cell preparations with a viability > 80% were used for experiments. The isolated cells were seeded on collagen type I-coated culture dishes at a density of 1.2·10^5 ^cells/cm^2^. Tissue samples from human liver resection were obtained from patients undergoing partial hepatectomy for metastatic liver tumour secondary to colorectal cancer. Tumour aggregates were resected including a safety margin within the normal tissue and visual inspections from the surgeon confirms that tumour-free liver tissue was obtained for cell isolation. Experimental procedures were performed according to the guidelines of the charitable state-controlled foundation Human Tissue and Cell Research, with the patient's informed consent [50], as approved by the local ethical committee. The day after isolation, the primary hepatocytes were cultivated for 2 days in Williams Medium E supplemented with 2 mm l-glutamine (Invitrogen), 100 nm dexomethasone (Sigma) and 1% penicillin ⁄ streptomycin (Invitrogen).

Huh7 cells were plated on 96-well plates coated with collagen type I-coated (BD Biosciences, Franklin Lakes, NJ) at approximately 30,000 cells/well in DME medium containing 10% Fetal Bovine Serum (FBS). After an overnight incubation, cells were starved for 4 hours, treated with inhibitors for 40 minutes, and then with stimuli. In each well different experimental conditions were imposed by introducing a combination of stimulus and inhibitor. Kinase inhibitors were used at concentrations sufficient to inhibit at least 95% of the phosphorylation of the nominal target as determined by dose-response assays in previous work [13]. The following concentrations were chosen: EGFR/Lapatinib (3 μM), EGFR/Erlotinib (1 μM), cMET/JNJ38877605 (1 μM), MEK/PD325901 (100 nM), PI3K/PI-103 (10 μM), p38 (100 nM), IKK/IMD (10 μM). After 40 minutes of incubation with the inhibitors, the cells were treated with saturated levels of 7 stimuli: IL1b (10 ng/ml), INS (2 μM), IL6, TGFA (100 ng/ml), TNFa (100 ng/ml), HGF (100 ng/ml), HER (100 ng/ml) in two separate plates for 5 and 25 minutes. The selection of these two time points was based on preliminary results published in [13], that identified 5 and 25 minutes as the optimal reporters of early phosphorylation activities. At the end of the treatment cell lysates were collected using standard lysate procedure [13]. Lysates from 5 and 25 minutes were pooled together in 1:1 ratio. The mixed lysate -that corresponds to an "average early signalling response"- was measured using the Luminex xMAP technology. Mixing cell lysates serves multiple purposes such as significant decrease of experimental cost and improvement of data quality [23]. The following phosphoprotein bead set from Bio-Rad were used: Akt (Ser473), CREB (Ser133), ERK1/2 (Thr202/Tyr204, Thr185/Tyr187), GSK3(Ser21/Ser9) Histone H3 (Ser10), HSP27 (Ser78), IκB-α (Ser32/Ser36), IR-β (Tyr1146), IRS-1Ser (Ser636/Ser639), JNK (Thr183/Tyr185), MEK1 (Ser217/Ser221), p38 (Thr180/Tyr182), p70S6K (Thr421/Ser424), STAT3 (Ser727), p90RSK (Thr359/Ser363), IGF-1R (Tyr1131).

Apart from the phosphoprotein signals, cytokine release was also measured from supernatants under the same experimental conditions. After incubating the cells with the inhibitors for 45 minutes, the same stimulus/inhibitor combinations were applied and following overnight incubation we removed the supernatant and measured the secretion of 33 cytokines. Among the 33 cytokines, 22 cytokines were shown an activity and were used in the subsequent data analysis. The cytokines used are: bFGF, Eotaxin, GMCSF, IFNγ, IL15, IL17, IL1b, IL1ra, IL2, IL4, IL6, MCP1, MIP1α, MIP1β, IP10, GROa, ICAM, GCSF, RANTES, TNFα, VEGF, SDF1. The 11 cytokines that were excluded are: IL9, IL10, IL12p70, IL13, IL5, IL7, IL8, PDFG, MIF, MIG, VCAM1.

### Computational Procedure

#### Data Processing and Linear Regression Analysis

Both signalling and response datasets were organized in data structures in the form of 5-D cubes using the DataRail software [35]; 4 of the dimensions of the cube correspond to the different experimental conditions (cell type, time point, stimuli treatment, inhibitor treatment) and the 5th to the measured readouts (response data and signalling data). The raw data for both response and signalling datasets were then normalized using a hill function filter and scaled to the range [0,1] as described previously [24] (See Additional File 3, S1 for an assessment of the proposed method's sensitivity to variables of the normalization procedure). The noise level of the assay has been estimated in [13] at the range of 166 fluorescent units, by considering the standard deviation of repeated measurements of unstimulated controls. The response matrix **Y^Res ^**(an m × k matrix representing m response component under k conditions) was then regressed against the signalling matrix **X^Sig ^**(an m × k matrix representing m intracellular signals under k conditions). The computed correlation matrix **W **is comprised by the correlation coefficients *w_i,j_*, where *i *is the index of response components (*i = 1..num_res*) and *j *the index of signals (*j *= 1..*num_sig*). The correlation coefficients *w_i,j_*, were then used as the stoichiometric weights of the non-signalling reactions in the Boolean framework that originates from a signal *j *and ends to a response component *i *(see also ILP formulation section and Additional File 3, S3 for an estimation of the proposed method's sensitivity to *w_i,j _*).

#### ILP Formulation

##### Non-signalling edges

The ILP formulation first used to optimise Boolean signalling pathways in [23], is extended herein to include response measurements. The main concept revolves around the minimisation of an objective function that represents the deviation between the experimental measurements and the values inferred from the network topology penalized by a function of the map's size. The ILP prunes the pathway by removing all edges that contradict the respective dataset, thereby minimizing the value of the objective function:(2)

where the summation is only over the relevant terms as described below.

Major addition to the formulation, compared to the one introduced in [23], consists the set *j_res _*= {1,...,ns_,res_} of response species, and the set *i*_*res *_= {1,...,n_*r,res*_} of edges linking signalling (*j*) with response (*j_res_*) nodes. The rest of the used symbols are as follows:

- , are weights set by the user,

- is the predicted value of species j in the experiment k,

- is the measured value of species j in experiment k,

- denotes the activation or not of reaction i in experiment k.

- *y_i_*, is a variable denoting whether a reaction is possible or not *y_i _*= 0 ∨ *y_i _*= 1.

- The term  corresponds to  and is the predicted value of response species *j_res _*in experiment k. It equals to the sum of all reactions *i_res _*leading to species *j_res _*weighted by , i.e., the Multiple Linear Regression weights. In other words the summation is only over the reactions *i *that lead to response species *j*.

Therefore, the first term of the objective function , corresponds to the measurement-prediction mismatch over all signalling species (*j*) and experimental conditions (*k*). Note that the summation is only taken over the species *j *that are measured in experiment *k*.

The second term , corresponds to the measurement-prediction mismatch over all response species (*j_res_*) and experimental conditions (*k*). The middle summation is over the response species *j *that are measured in experiment *k*. The inner summation is over the reactions *i *that lead to response species *j*.

The third term , corresponds to the penalty imposed by the map size. For a complete reference to the original formulation see [23]. Here we will only discuss the extra constraints regarding the response species. An extensive assessment of the proposed method's performance under different values of  and  is illustrated in Additional File 3, S3.

Concerning the term , assuming ,  corresponds to the scaled measurement-prediction error. Let the minimal and maximal total yields (and thus expected measurements) of the species be given by

We want to minimize the weighted sum of the absolute differences . Assuming that the measurement is consistent with the weights, we would have  which would give . However, this cannot always be assumed and therefore we take the more general case that

We can thus scale as

to

and obtain the desired range, i.e., . To ensure linearity we impose two inequality constraints, which are equivalent for ,

The above constraints complete the formulation.

##### Solution pool

As aforementioned, the objective function (1) consists of three major terms, namely  and  which are related to the goodness of fit, and  which penalises the size of the pathway. The need for the third term arises from the fact that there are many solutions fitting the measurements equally good. To reduce the number of optimal solutions the size of the pathway is also minimised. However, the biological significance underlying the minimisation of the pathway's size is not evident. Thus, we introduce a tolerance of the global minimum and harvest 100 solutions lying within this tolerance. This modification allows us to consider a solution pool instead of a single solution. The frequency of each edge in the solution pool, expresses a level of confidence in the presence or absence of the respective edge in the optimal pathway. By taking into account suboptimal solutions we are sure to capture relations between the signalling cascades, and their probability of occurrence, that we might otherwise miss.

#### Error Calculation

The error is defined as the deviation between experimental and simulated values using the following formula:

, is the predicted value of species j in the experiment k,

, is the measured value of species j in experiment k,

## Competing interests

The authors declare that they have no competing interests.

## Authors' contributions

INM and LGA conceived the project. AM conceived and implemented the ILP formulation and its extension to response measurements. AM wrote the largest portion "Computational Procedure" subsection in the "Materials and Methods" section. LGA and INM wrote the remainder of the manuscript. TSW developed the isolation protocols and provided the hepatocytes. LGA and DEM performed the experiments. INM run the code and analyzed the data. AM and TSW edited the manuscript. All authors approved the final manuscript.

## Supplementary Material

Additional file 1 **Signalling dataset**. Dataset in MIACA format (phosphorylation data) that were produced and used in this manuscript (see also figure 2). Click here for file

Additional file 2 **Response dataset**. Dataset in MIACA format that were produced and used in this manuscript (see also figure 2). Click here for file

Additional file 3 **Supplementary Materials and Methods**. "Supplementary Materials and Methods" include further information about the proposed methodology, such as, i) data normalisation procedure used, ii) comparison with an alternative 2-step Multiple Linear Regression method, iii) a detailed model assessment and iv) comparison of simulation runs for Huh7 and Normal cells. Click here for file
