# Supplementary material for: Combined logical and data-driven models for linking signalling pathways to cellular response
Source: BMC Syst Biol. 2011 Jul 5;5:107. doi: 10.1186/1752-0509-5-107 (PMC3145575; doi:10.1186/1752-0509-5-107)
Supplement: Additional file 3 — Supplementary Materials and Methods. "Supplementary Materials and Methods" include further information about the proposed methodology, such as, i) data normalisation procedure used, ii) comparison with an alternative 2-step Multiple Linear Regression method, iii) a detailed model assessment and iv) comparison of simulation runs for Huh7 and Normal cells. [file 1752-0509-5-107-S3.DOC]

**Supplementary Information for**

**Combined logical and data-driven models for linking signaling pathways to cellular response**

Ioannis N. Melas^1*^, Alexander Mitsos^2*^, Dimitris E. Messinis^1^, Thomas S. Weiss^3^, Leonidas G. Alexopoulos^1 §^

^1^ Dept of Mechanical Engineering, National Technical University of Athens, 15780 Zografou, Greece

^2^ Dept of Mechanical Engineering, Massachusetts Institute of Technology, Cambridge, MA 02139, USA

^3^ Center for Liver Cell Research, Department of Pediatrics and juvenile Medicine, University Medical Center Regensburg, Regensburg, Germany

^*^These authors contributed equally to this work

^§^Corresponding author

**This file includes:**

S1: Data Normalisation 2

S2a. Construction of a 2-step Multiple Linear Regression (MLR) model 5

S2b. Comparison of proposed ILP-MLR hybrid Model to 2-step MLR 8

S3. Model Assessment – sensitivity of the proposed approach to experimental

design, data, generic topology, linking weights and **a*^k^_jr_*** constants 9

S4. Impact of response measurements on pathway optimisation 15

S5. Comparison of simulation runs for Huh7 and Normal cells 16

**S1. Data Normalisation**

In this paper we apply a normalisation of raw data from 0 to 1 as described previously (Saez-Rodriguez et al, 2009) by considering 1) the percent change from basal to stimulated state, 2) the experimental noise, 3) the upper limit due to the saturation of the assay (~30,000), and 4) the basal level at time zero. The most important parameter is the activation threshold where a signal is considered “active” and should be mapped to a value greater than 0.5 in the 0/1 logic (a signal greater than 0.5 will favour a Boolean value of 1 on the optimisation scheme in order to minimise the experimental/computational mismatch). To assess activity, the stimulated state (average of 10 and 30 mins for phosphoproteins, and 24 hours for cytokine release) is compared to its unstimulated state (basal levels at time zero). In previous work (Saez-Rodriguez et al, 2009) the default activation threshold value was set to 2, which implies that a two-fold increase compared to unstimulated state is considered an active signaling event. Here, in order to identify an optimal value for our particular dataset, we optimised the generic pathway for several different threshold values ranging from 1.1 fold increase (a 10% increase is considered significant) to 7 fold increase (a 700% increase is considered significant) and we look into the behavior of two parameters a) the number of edges conserved by the optimisation algorithm (Additional File 3, Figure S1) and b) the optimisation error (Additional File 3, Figure S2).

*Number of edges conserved:* For each value of fold increase we documented the number of edges that are conserved by the ILP algorithm when compared with the generic topology. Additional File 3, Figure S1 shows a logarithmic decrease of conserved reactions as the fold change threshold increases. For low threshold values (1.10🡪 1.40) almost all the original edges are conserved, since even the slightest increase of the signal is considered to be significant. The resulting pathway very much resembles the generic topology. Then a decrease of the edges number is observed as the threshold increases. For thresholds in the range of 1.50🡪2.5, the number of conserved edges lies in (60🡪80). As the threshold increases beyond 2.5, the number of reactions drops substantially until it reaches 0 at the threshold of 7

*Optimisation Error:* Additional File 3, Figure S2 presents the total error between topology predictions and experimental data for “Activity thresholds” that range from 1.1 to 7. The error curve dictates that the optimisation results are lower at threshold values between 1.8 and 2.0 where the minimum optimisation error is ~18%. Even though our point is not to find a threshold to the ILP formulation that does not cause problem, the Additional File 3, Figure S2 can help us understand how optimisation algorithm performs and define ranges for optimal thresholds. For very low activity thresholds (left side of the curve) the normalised experimental data show significant activity and thus the dataset is too noisy for the algorithm to optimise the map, because of many active signals contradicting one another. As a result, the optimisation algorithm cannot find a pathway that satisfies most of the signaling activities and the optimisation error is high. For large activation threshold values (~6 fold increase, right side of the curve in Additional File 3, Figure S2) the normalisation algorithm perceives many signals that are truly significant (a 5 fold increase is considered noise) as unstimulated signals. As a result, the respective edges are removed although they are functional and the total optimisation error is increased (see threshold values above 2.0 in Additional File 3, Figure S2).

Taking the above observations in consideration, a threshold between 1.8 and 2.0 should be used where the algorithm performs better.


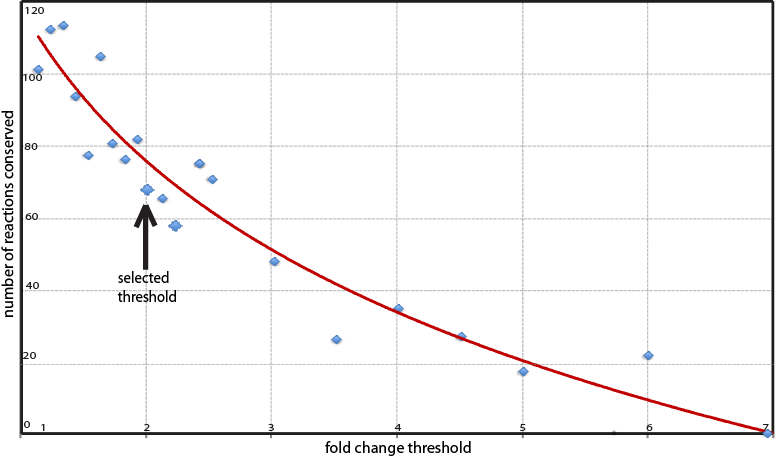


*Figure S1. “Activity threshold” or “fold change threshold” is an important parameter for comparing Boolean pathways with experimental data. The number of edges conserved after optimisation (y a-axis) depends strongly on the threshold set for assuming protein activity. Activity thresholds (x-axis) range from 1.1 (a 10% increase between basal level and stimulated level is considered active) and 7 (700% increase is considered active).*


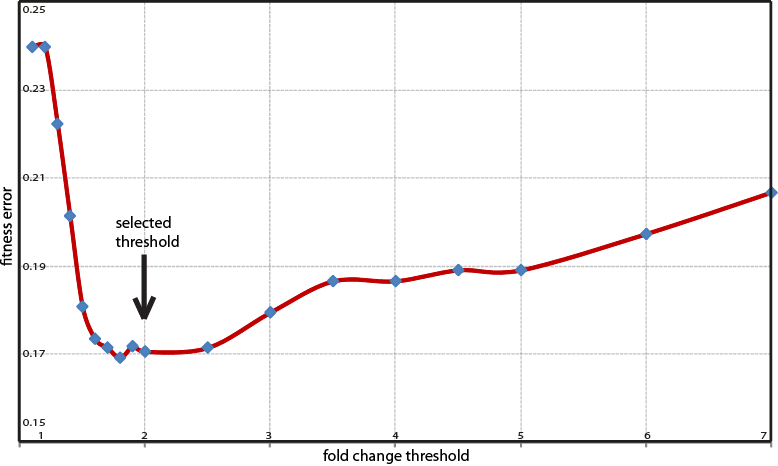


*Figure S2. Total error after optimisation (Y-axis) as a function of “Activity threshold” (X-axis).*

**S2a. Construction of a 2-step Multiple Linear Regression (MLR) model**

The performance of our hybrid ILP-MLR approach is compared against a 2-step MLR approach that correlates stimuli and inhibitors to the measured phosphoproteins in a simple regression manner *{Alexopoulos et al. Mol Cell Proteomics 2010}*. Phosphoprotein signals are then linked via MLR to the cytokine releases.

An ***(n x k)*** matrix ***X^cue^*** is put together, where ***n*** equals to the number of stimuli and ***k*** equals to the number of experimental conditions. ***X^cue^(i,j) = 1*** implies stimulus ***i*** is included in experiment ***j,*** else ***X^cue^(i,j) = 0.*** In similar fashion we introduce an ***(m x k)*** matrix ***X^inh^*** where ***m*** equals to the number of inhibitors and ***X^inh^(i,j) = 1*** if and only if inhibitor ***i*** is included in experiment ***j.*** Furthermore, an ***(s x k)*** matrix ***Y^sig^*** is put together, where ***Y^sig^(i,j)*** equals to the measured value of signal ***i*** in experiment ***j.***

The ***(s x n)*** matrix ***W^cue^*** is defined such that, ***Y^sig^ = W^cue^*** •***X^cue^ . W^cue^*** is computed via Linear Regression using Matlab. The residual matrix, ***RES=Y^sig^ – W^cue^*** •***X^cue^*** is then expressed as ***RES=W^inh^***•***X^inh^. W^inh^*** is computed via Linear Regression. Matrices ***W^cue^*** and  ***W^inh^*** express the effects of each cue and inhibitor on the measured phosphoproteins.

Subsequently, ***Y^cyt^*** is introduced, consisting of the cytokine release measurements such that ***Y^cyt^(i,j)*** corresponds to the value of cytokine release ***i,*** in experiment ***j***. Matrix ***W^sig^*** is defined such that ***Y^cyt^=W^sig^***•***Y^sig^ ,*** where ***Y^sig^*** was defined previously.

Using the 2-step MLR approach, we performed the following tasks:

1. Correlate each stimuli and inhibitor with the intracellular protein activity (measured phosphoproteins )
2. Correlated phosphoprotein activities with the cytokine release
3. Construct an executable signal transduction model, able to predict cellular response upon external perturbation (in the form of stimuli and inhibitors).

Additional File 3, Figure S3 features the connectivity patterns underlying Huh7 data; the edge thickness corresponds to the absolute value of MLR weights and edge colour corresponds to weights’ sign. Simulation of the MLR model, upon external perturbation, can be performed by compiling the ***X^cue^*** and ***X^inh^*** matrices full of 0’s and 1’s in a way that reflects the experimental conditions, then simulation results on the phosphoprotein and cytokine release level are obtained: ***Y^sig^ = W^cue^*** •***X^cue^+ W^inh^***•***X^inh^, Y^cyt^=W^sig^***•***Y^sig^ .*** Simulation runs are rounded to 1 or 0, (denoting activation or not respectively) for easier comparison with our Boolean-based ILP approach and illustrated in Additional File 3, Figure S4. An evaluation of the 2-step MLR approach can be obtained by computing the measurement – prediction mismatch via the following formula :

,

- $x_{j}^{k}$, is the predicted value of species j in the experiment k,
- $x_{j}^{k,m}$, is the measured value (m) of species j in experiment k,


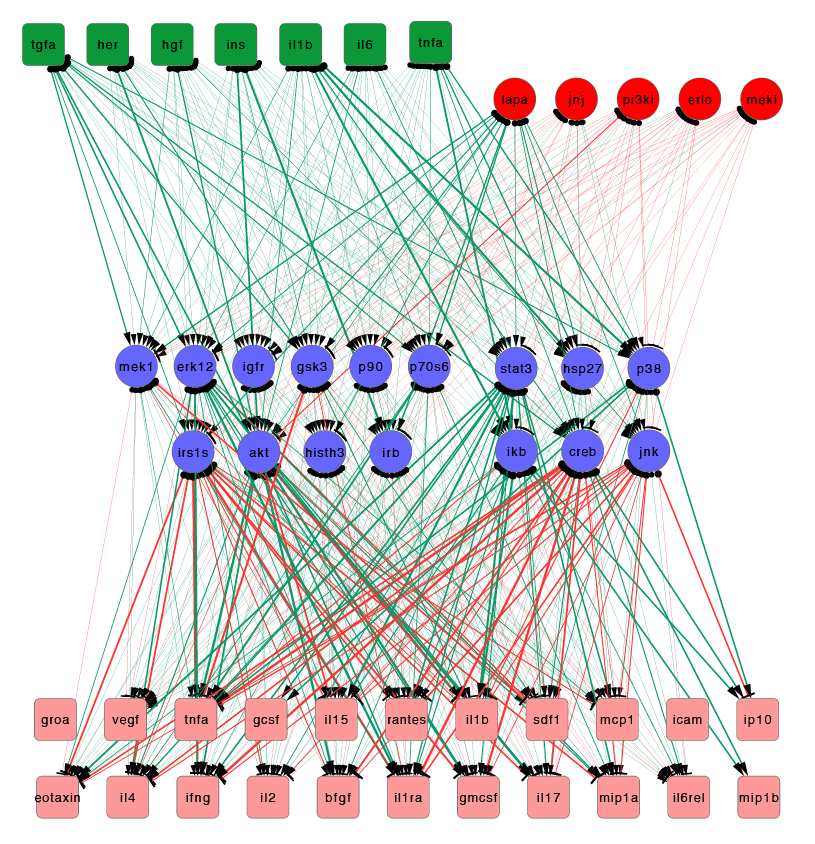


*Figure S3. 2-step MLR model. Cytokines and inhibitors are linked to phosphoprotein signals via weights obtained by MLR. In a similar fashion, phosphoproteins are linked to cytokine releases. Edges thickness and opacity corresponds to the absolute value of the respective weights. Edges colour corresponds to interaction type (green=activation, red=inhibition).*

**
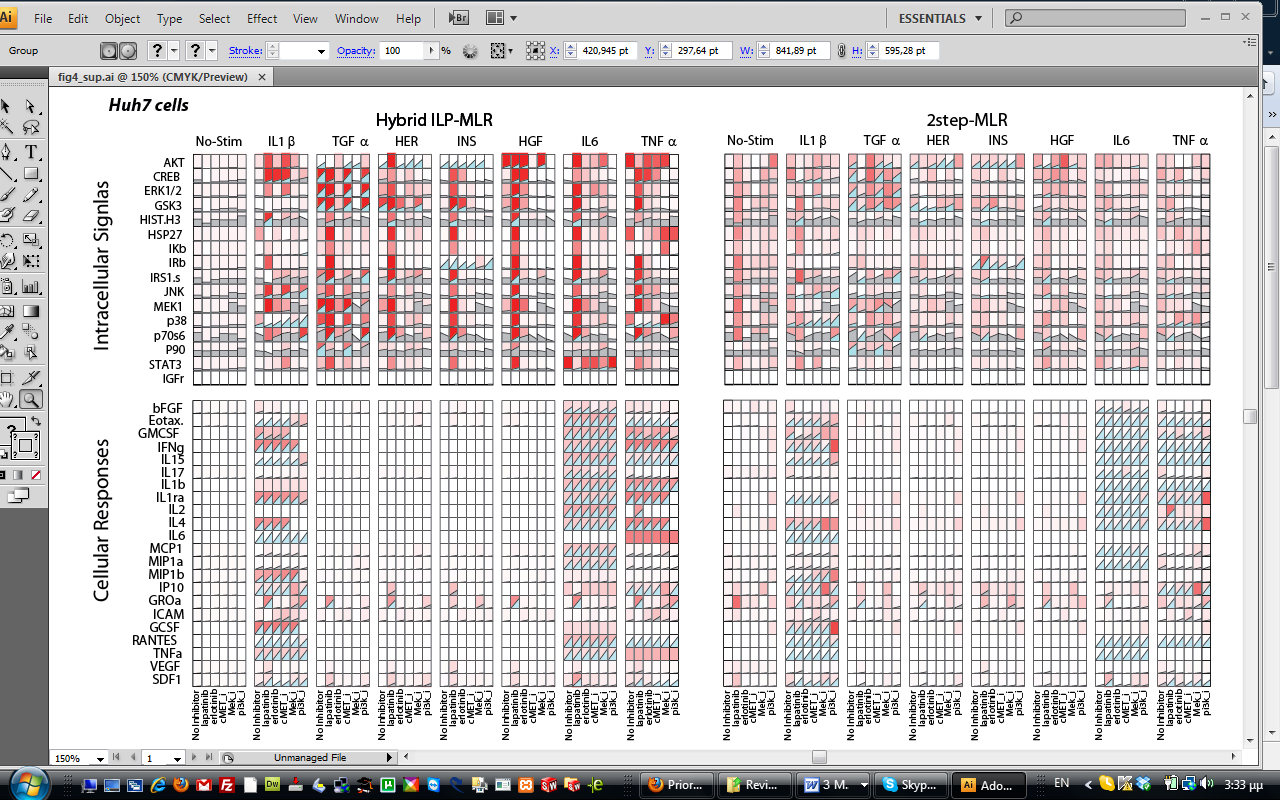
**

**
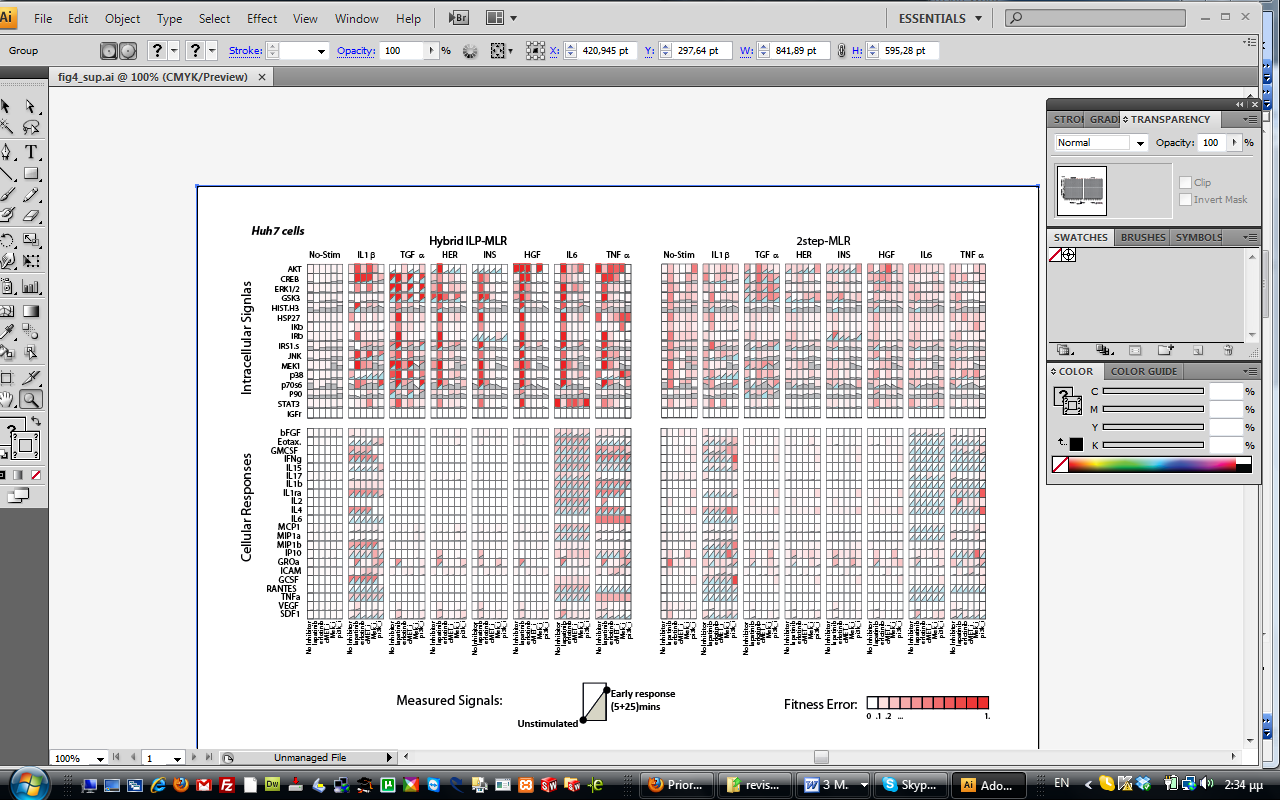
**

*Figure S4. Simulation results of the Hybrib ILP-MLR (left panel) and the 2-step MLR (right panel) models. The intensity of the red background corresponds to the fitness error.*

**S2b. Comparison of proposed ILP-MLR hybrid Model to 2-step MLR.**

We attempt to compare the 2-step MLR approach presented above with our proposed hybrid ILP-MLR methodology in terms of i) data fitting and representation, ii) biological significance and relevancy of the results.

*Data fitting and representation*

The 2-step MLR approach performed best out of the two methods regarding data fitting and representation as estimated by the measurement – prediction mismatch (12% for 2-step MLR, 18% for the hybrid ILP-MLR formulation). It is expected for a data driven approach to perform better at data fitting and representation than a topology driven approach since no extra constraints are introduced from having to comply with a priori knowledge of the signaling network’s connectivity. For instance, different activation patterns of MEK1 (or ERK12 and CREB) across various EGFR ligands such as TGFa, HER, HGF and even INS (although not an EGFR ligand) is only feasible in a data driven approach, since a topology driven approach will have to comply with a restrictive generic topology, such as the one used here, where TGFa, HER and HGF signal through overlapping pathways. Therefore if TGFa activates MEK1 so does HER and HGF. A topology driven approach, such as the ILP will either conserve the whole branch (RAS 🡪 RAF1 🡪 MEK1 🡪 ERK12) thus activating the respective signals under all EGFR ligands, or remove the entire branch. The optimal solution is the one that least increments the measurement – prediction mismatch, however, both alternatives lead to an increase in the fitness error. Additional File 3, Figure S4 features many examples like the one just described such as: different activation patterns for HSP27 and CREB under IL1b and TNFa, differential activation of GSK3 under TGFa, HER, HGF and INS.

*Biological significance and relevancy of the results*

Incorporation of a priori knowledge in the form of a generic pathway assists in obtaining biologically relevant and significant results, since the generated model need not only fit experimental data adequately, but also comply with literature. The proposed ILP-MLR hybrid approach by taking into account a priori knowledge of proteins connectivity guarantees biologically interpretable results. Moreover, the activation state of latent nodes can be inferred given the activation state of others, while 2-step MLR ignores any intermediate nodes. Further comparison of data driven versus topology driven methods goes beyond the purpose of this paper, the reader may find an elaborate characterization of these classes of methods in *{Aldridge et al. Nat Cell Biol 2006}.*

**S3. Model Assessment: Sensitivity of the proposed approach to experimental design, data, generic topology, linking weights and $a^{k_{j_{r}}}$constants**

For better assessment of the proposed methodology, its sensitivity to changes in

1. experimental design

2. measured data

3. generic topology

4. linking weights

5. $a^{k_{j_{r}}}$ constants

is assessed, in terms of i) **remaining fitness error** and ii) **topology alterations.**

The ***“*remaining fitness error”** corresponds to the measurement-prediction mismatch in the pathway after the optimisation and it is evaluated by the following formula:

,

- $x_{j}^{k}$, is the predicted value of species j in the experiment k,
- $x_{j}^{k,m}$, is the measured value (m) of species j in experiment k,

**“Topology alterations”** aim to identify the differences between i) the pathway optimised with a reduced/altered dataset and ii) the pathway optimised with the full dataset. The differences are captured by comparing simulation runs, following the formula:

,

- $x_{j}^{k}$, is the predicted value of species j in the experiment k, full-dataset optimised pathway

$x_{j}^{k,r}$, is the predicted value of species j in experiment k, reduced-dataset (r) optimised pathway

***S3.1. Sensitivity to changes in the experimental design***

The phosphoproteomic dataset consists of 48 experimental conditions (8 stimuli including the no-stimulus treatment, times 6 inhibitors, including the no-inhibitor treatment) and 16 signals; the response dataset consists of the same experimental conditions and 22 signals. In this part of model assessment, we exclude random subsets of the 48 experimental conditions and monitor how the ILP algorithm performs. 10 in-silico experiments are carried out, leaving out 5%, 10%, 15%, 20%, 25%, 30%, 35%, 40%, 45% and 50% of the experiments.

It becomes apparent that the greater the subset of experimental conditions left out of the optimisation procedure is, the poorer the fit we obtain becomes, in what seems to be a linear fashion (R^2^=0.88995) (Additional File 3, Figure S5). Finally, having excluded 50% of the experimental conditions, the remaining fitness error reaches 35% (almost two times the fitness error of the full-dataset optimised pathway).

*Figure S5. Sensitivity of proposed methodology to experimental design. First curve (blue points) corresponds to remaining fitness error upon excluding subsets of the experimental conditions. Second curve (red points) corresponds to topological alterations between the full-dataset optimised map and reduced-dataset optimised map.*

***S3.2 Sensitivity to data deterioration***

In this part of model assessment, we scramble random subsets of the original phosphoproteomic and response datasets. 5%, 10%, 15%, 20%, 25%, 30%, 35%, 40%, 45% and 50% of the total datapoints are substituted with random numbers in (0,1). Like before, the remaining fitness error and topology alterations are computed via formulae (1), (2) and the results are plotted in Additional File 3, Figure S6. Scrambled data are characterized by internal conflicts (e.g., activated values in no-stimuli experiments), the ILP algorithm cannot emulate this behavior, resulting in increasing fitness error with limited alterations in the topology of the optimised pathway.

*Figure S6. Sensitivity of proposed methodology to data scramble. First curve (blue points) corresponds to remaining fitness error upon substitution of datapoints with random numbers in (0,1). Second curve (red points) corresponds to topological alterations between the full-dataset optimised map and scrambled-dataset optimised map.*

***S3.3 Sensitivity to changes in the generic topology***

Herein, we assess the sensitivity of the model to changes in the generic topology. In similar fashion to previous cases, subsets of reactions (2%, 4%, 6%, 8%, 10% of the generic pathway) are substituted with random connections. Scrambled pathways are optimised using phosphoproteomic and cytokine release datasets and the remaining fitness error and topology alterations are plotted in Additional File 3, Figure S7. As expected the generated models are very sensitive to changes in the generic topology, even a single reaction removed can cause drastic changes in the model behavior. For instance, removal of EGF🡪EGFR prevents the ILP from successfully fitting all experiments where EGF is introduced. Deviation of simulation runs also increases with increasing number of scrambled reactions, but to a smaller extent, implying that removed reactions have disrupted the signal transduction and no alternative paths exist.

*Figure S7. Sensitivity of proposed methodology to changes in the generic topology. First curve (blue points) corresponds to remaining fitness error upon substitution of reactions with random connections between the species. Second curve (red points) corresponds to topological alterations between the map optimised using the original generic topology and map optimised using the scrambled generic topology.*

***S3.4 Sensitivity to changes in modeling decisions:*** $w_{\text{cr}}$

Weights obtained via MLR are used to link intracellular signal transduction pathways with cellular response (herein cytokine releases), in a consistent, integrative model. MLR generates connections from each of the measured phosphoprotein to every cytokine released, resulting in a total of 352 reactions. However, most of the respective weights are very close to zero, suggesting very little (if any) effect on the released cytokines. To increase computational efficiency, only reactions with weights of absolute value greater than $w_{\text{cr}}=0.2$ are considered in the optimisation procedure (see “Methods” section in the paper). Herein, we test model’s sensitivity to this arbitrary threshold by running the optimisation procedure for a range of values (0, 0.1, 0.2, 0.3, …, 2.0). The remaining fitness error for the whole model as well as the non-signaling part alone is plotted in Additional File 3, Figure S8. The arbitrary threshold ($w_{\text{cr}}$ ) has little effect on the signaling part of the model. Concerning the non-signaling part, increasing threshold values leads to drastic increase in the fitness error. For small values (0.0, 0.1, 0.2) fitness error is relatively stable (0.082 🡪 0.10), however, for values greater than 0.3 fitness error increases significantly.

*Figure S8. Sensitivity of proposed methodology to changes in $w_{\text{cr}}$. First curve (blue points) corresponds to remaining fitness error for the whole model as a function of the arbitrary threshold $w_{\text{cr}}$. Second curve (red points) corresponds to remaining fitness error for the non-signaling part of the model as a function of the arbitrary threshold $w_{\text{cr}}$.*

***S3.5 Sensitivity in modeling decisions:*** $a^{k_{j_{\text{res}}}}$

In this part of model assessment we examine the model sensitivity to changes in the user defined constants $a^{k_{j_{\text{res}}}}$. The ratio , determines how the ILP prioritizes signaling over cytokine release measurements. Throughout the analysis presented in this paper we have selected . Herein we test a range of values (10,20,40,76.1905,100,120,150,200,250,300,350,400,450,500) for $a^{k_{j_{\text{res}}}}$, and monitor changes in the remaining fitness error for the whole model, as well as the non-signaling part alone (Additional File 3, Figure S9).$a^{k_{j_{\text{res}}}}$has little effect on the behavior of the model as a whole, since no conflicts exist between the phosphoprotein and cytokine release datasets. Concerning the non-signaling part of the model, small values of $a^{k_{j_{r}}}$ ($a^{k_{j_{r}}}=10.0$) imply the cytokine release measurements have little weight on the objective function, and the respective reactions are excluded from the solution leading to an increase in fitness error of the response dataset. For greater values, error drops significantly and stabilizes to ~10%. The value implies term equals to , thus, cytokine release data and signaling data are treated equally.

*Figure S9. Sensitivity of proposed methodology to changes in $a^{k_{j_{r}}}$. First curve (blue points) corresponds to remaining fitness error for the whole model as a function of $a^{k_{j_{r}}}$. Second curve (red points) corresponds to remaining fitness error for the non-signaling part of the model as a function of $a^{k_{j_{r}}}$.*

**S4. Impact of response measurements on pathway optimisation**

To assess the effects of response measurements on pathway optimisation we optimised canonical pathways with the Huh7 dataset on three different ways: a) using the signaling data (top panel of Additional File 3, Figure S10) as described previously (Mitsos et al, 2009), b) using response data (cytokine secretion, bottom panel of Additional File 3, Figure S10) and c) using both signaling and response data. Pathway results are presented in Additional File 3, Figure S10 (the optimisation with all data are the same as in Additional File 3, Figure S10 and is presented here for comparison reasons).

When optimised with response data only, we find that pathways which are not connected with any cytokine releases (ex. INS, HGF) are removed during the optimisation process. On the other side, because of HuH7 cells release cytokines only upon IL6, TNFa and IL1b stimulation, the ILP algorithm conserves those paths and connects them to the cytokine releases via the nodes that are highly correlated to the release such as IKB, STAT3, and P38. An interesting observation is the IL6🡪STAT3🡪Cytokine release. Despite the fact that the IL6🡪STAT3 pathway had been removed when only signaling data were used (because STAT3 activation is below threshold as shown in IL6-induced STAT3 data in Additional File 3, Figure S10) the same pathway is conserved when response data are used. The reason of that seemingly contradictory observation is because the small changes of STAT3 activation correlate well with a large number of cytokine releases and thus, the MLR algorithm attributed large correlation weight on the corresponding non-canonical edges. Subsequently, the ILP formulation conserves those non-canonical edges by connecting them to the respective stimulus. Thus, response data further constrain the optimisation formulation and take advantage of the power of statistical analysis that identifies connections between signals and cellular response.


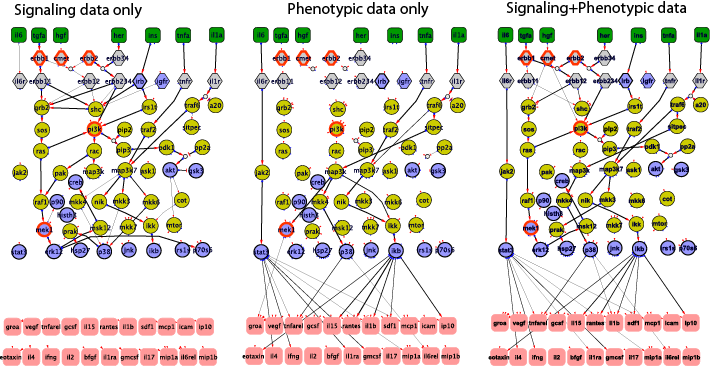


*Figure S10: Optimisation of the canonical pathway using either (a) signaling data, (b)response data, and (c) both signaling and* response *data.*

**S5. Comparison of simulation runs for Huh7 and Normal cells**

Differences regarding the signal transduction mechanisms of Primary and Huh7 cells are illustrated in “Results” section of this paper with emphasis given on connectivity patterns of the optimised topologies. Herein we demonstrate how these differences are reflected on simulation runs of the generated models. Additional File 3, Figure S11, features the signed matrix:


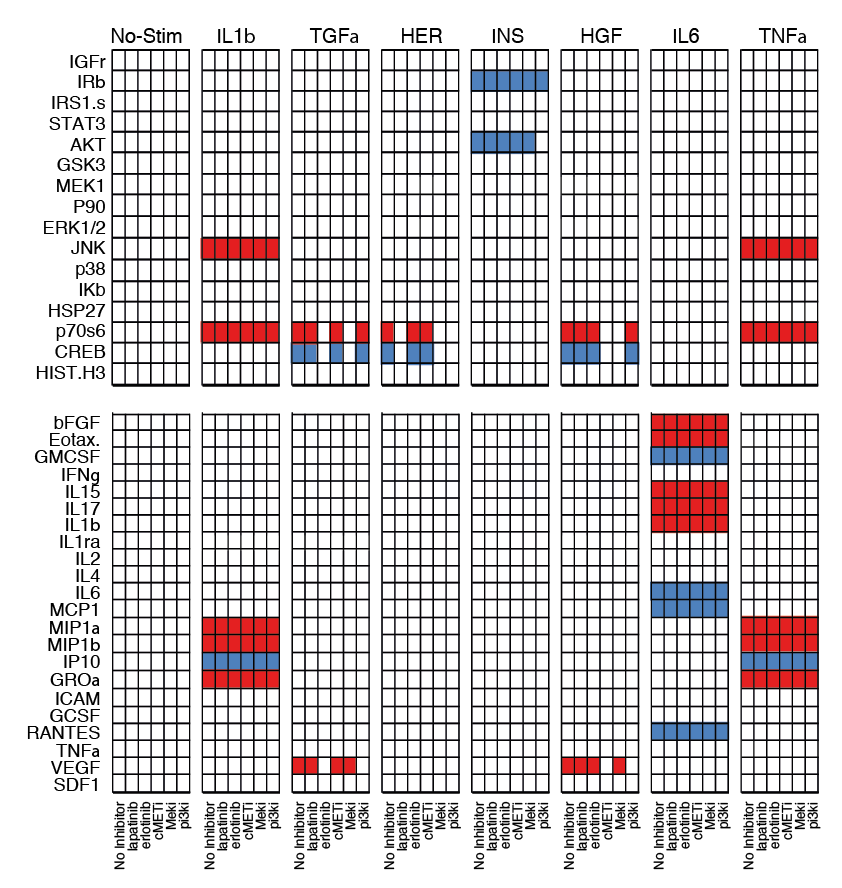


*Figure S11. Comparison of simulation runs for Huh7 and Normal cells. Negative values ( Y^NORMAL^ < Y^HUH7^ ) are plotted in blue, positive values ( Y^NORMAL^ > Y^HUH7^ ) are plotted in red.*

The phosphoproteins part of Additional File 3, Figure S11 reveals differential activation patterns for JNK, p70s6 and CREB signals, in addition to activation of the INS pathway present only in Huh7 cells. Concerning the prediction of cytokine releases, Primary cells release bFGF, Eotaxin, IL15, IL17, IL1b, MIP1a, MIP1b, GROa, and VEGF in contrast to Huh7 cells that release GMCSF, IL6, MCP1, IP10 and RANTES. Simulation runs presented here are in accordance with the connectivity patterns described in the main part of the paper.
